# DNA damage of peripheral blood lymphocytes as a dual biomarker: Diagnostic and treatment response in woman breast cancer patients

**DOI:** 10.1177/18758592241308748

**Published:** 2025-03-20

**Authors:** Ana Rita Guedes, Jorge Pinto Soares, Renato Cunha, Amélia Maria Silva, Isabel Gaivão

**Affiliations:** 1 Animal and Veterinary Research Center (CECAV) and Department of Genetics and Biotechnology, University of Trás-os-Montes and Alto Douro (UTAD), Vila Real, Portugal; 2 Associate Laboratory for Animal and Veterinary Sciences (AL4AnimalS), UTAD, Vila Real, Portugal; 3 Department of Biology and Environment (DeBA) and Centre for Research and Technology of Agro-Environmental and Biological Sciences (CITAB), UTAD, Vila Real, Portugal; 4 Research Centre of Sports, Health, and Human Development (CIDES), UTAD, Vila Real, Portugal; 5 Local Health Unit of Trás-os-Montes and Alto Douro, Vila Real, Portugal

**Keywords:** breast cancer, DNA damage, treatment, human lymphocytes, biomarker

## Abstract

**Background:**

Breast cancer is the leading malignancy among women and the lack of ideal early biomarkers hampers diagnosis and treatment monitoring. Genomic instability, central to breast cancer development, makes DNA damage a potential biomarker for these purposes.

**Objective:**

This study aims to evaluate the predictive value of DNA damage for diagnosis, and treatment monitoring in breast cancer, with CA 15-3, a conventional cancer biomarker, included for comparison to assess the added value of DNA damage measurement.

**Methods:**

DNA damage was measured in peripheral blood lymphocytes of 58 breast cancer patients, and 31 healthy controls, employing comet assay, both before and after treatment. Serum CA 15-3 levels were assessed at the same time points for comparison.

**Results:**

DNA damage levels were significantly higher in cancer patients compared to healthy controls, with the most elevated levels observed in patients with advanced-stage disease, irrespective of age, sex, lifestyle, or genetic status. Post-treatment assessments showed a significant rise in DNA damage. In comparison, CA 15-3 showed less consistent relevance for diagnostic and monitoring.

**Conclusions:**

This study underscores the greater potential of DNA damage as a consistent and reliable biomarker for breast cancer, with CA 15-3 providing complementary but less consistent data for clinical decision-making.

## Introduction

Breast cancer is the most prevalent malignancy worldwide, with an increasing incidence in recent years, according to the World Health Organization (WHO). It is the most diagnosed cancer in women, representing 11.7% of all cancer cases,^1^ and it is also the second cause of cancer-related deaths among women.^2^ The rise in the incidence of breast cancer can be attributed to increased exposure to risk factors and the heterogeneity of the disease, which limits early diagnosis and treatment efficacy.^3^ Despite advancements in early detection programs and effective therapies, conventional methods such as mammograms and biopsies have limitations, including false positives/negatives and challenges in detecting early-stage cancers.^4,5^ Cancer antigen 15-3 (CA 15-3), a glycoprotein produced and overexpressed in breast tumor tissue, plays a crucial role in assessing the effectiveness of treatment and identifying potential relapses at more advanced stages,^6,7^ but it also has limitations, particularly in the early stages of the disease, underscoring the necessity of a more comprehensive approach.

Factors contributing to breast cancer development include hormones,^8^ obesity,^9^ and environmental exposure.^10^ However, a family history of breast cancer is the most significant risk factor, though only 5-10% of all cancer cases have a strong inheritable component.^11^ Hereditary breast cancer is often associated with genomic alterations, such as mutations in DNA repair genes like BReast CAncer gene 1 (*BRCA1*) and BReast CAncer 2 (*BRCA2*), which are involved in repairing DNA double strand breaks (DSB), predispose individuals to breast cancer.^12^ High levels of DNA damage and deregulated repair mechanisms are key molecular events in cancer pathogenesis,^13,14^ as DNA damage occurs spontaneously due to environmental and endogenous agents.^15^ Persistent basal DNA damage may indicate heightened exposure to carcinogens and impaired DNA repair,^16^ which cannot be fully explained by genetic alterations alone. Increasing experimental evidence suggests that repair pathways have an estimated heritability of approximately 48-75%,^17^ with the remaining variation attributed to external factors.^18,19^ Hence, it is important to clarify the role of fluctuations in DNA damage levels in the development of cancer, particularly in cases where repair pathways are not mutated.

Since most cancer therapies are based on damaging DNA, genome maintenance is also important for a therapeutic response. Although surgery is the primary treatment, several patients receive adjuvant chemotherapy and radiotherapy to destroy any remaining cancer cells.^20^ Genotoxic anticancer drugs and ionizing radiation target the sensitivity of rapidly dividing cancer cells, inducing DNA damage, like single strand breaks (SSB), base alterations and DSB, which overcome DNA damage response (DDR) machinery, leading to apoptosis.^21,22^ Breast cancer therapy uses various chemotherapy drugs,^23^ including anthracyclines, such as doxorubicin,^24^ alkylating agents,^25^ and platinum agents, like cisplatin and carboplatin.^26^ Additionally, mitotic inhibitors, like docetaxel and paclitaxel are also employed.

Considering the important role of DNA instability in cancer, recent research has explored DNA damage and DDR as potential biomarkers for cancer diagnosis and treatment response.^27–29^ The comet assay has been widely used to assess DNA damage in tumor cells,^30–32^ with studies showing higher DNA damage levels in breast cancer patients compared to healthy individuals.^33,34^ Nevertheless, the reliability of measuring DNA damage levels in circulating lymphocytes for diagnosis and monitoring breast cancer remains a controversial issue.

The main objective of this study was to investigate whether assessing basal levels of DNA damage in peripheral blood lymphocytes (PBL) can act as a useful biomarker of DNA instability in sporadic and hereditary breast cancer monitoring and treatment response. Additionally, we aimed also to investigate the correlation between DNA damage levels and disease stage, as well as with the serum CA 15-3 levels, and to assess the effects of each type of treatment on DNA damage levels.

## Materials and methods

### Patients and clinical data

Blood samples were obtained from fifty-eight cancer patients, in the period from January 2019 to November 2022. The eligibility criteria for this study were as follows: women with a newly diagnosed primary breast cancer, who had not undergone any previous treatment that could damage DNA, suitable for treatment with chemotherapy (CT), radiotherapy (RT), or both (C-RT). Breast cancer patients who received chemotherapy were treated with anthracyclines, cyclophosphamide (alkylating agent), platinum agents, docetaxel, and paclitaxel. Blood samples were collected at two distinct time points: before treatment and, 4-6 months after starting treatment administration. Patients’ clinical and biographical data was obtained from their clinical processes. This included the stage of disease at diagnosis, drugs used in treatment, age, sex, and lifestyle habits such as alcohol consumption and smoking habits. To evaluate the hereditary susceptibility to cancer in younger patients and/or patients with a family history of hereditary cancer, the oncologist requested a genetic study using next-generation sequencing (NGS) panels, according to current clinical practice guidelines.^35^ Furthermore, we assessed the levels of PBL and CA 15-3 circulating levels, at each time point. Control subjects were obtained from non-oncologic donors with a negative mammogram performed within the previous year. Healthy participants had no exposure to potentially harmful chemicals, except for those encountered from environmental sources. No additional selection criteria were applied.

### Ethical approval

This study was approved by the Ethic Committee of the Local Health Unit of Trás-os-Montes and Alto Douro (number 495/2018 C.A.), Vila Real, Portugal, and each patient gave a written informed consent. It complies with The Code of Ethics of the World Medical Association (Declaration of Helsinki), printed in the British Medical Journal (18 July 1964).

### Blood collection and PBL's isolation

As mentioned, blood samples were collected from each patient before treatment (t0) and after 4-6 months from the beginning of treatment (t1) to evaluate the impact of anti-neoplastic drugs and radiation as DNA-damaging agents. Although the exact timing varied slightly between patients due to individualized treatment schedules, all patients received the same number of treatment cycles, ensuring consistency in exposure. Approximately 3 mL of blood was taken from each cancer patient at each time point by venous puncture. The controls, meanwhile, underwent a single collection using the same method. The blood was collected into ethylenediaminetetraacetic acid (EDTA) tubes and kept refrigerated (at 4°C) until processing. PBL were isolated by gradient density centrifugation (30 min, 800 ×*g*) using 3 mL of Histopaque 1077 (Sigma-Aldrich, St Louis, MO, USA). Then, the cloudy band above the density gradient medium containing PBL was transferred to a centrifuge tube containing 9 mL phosphate buffered saline (PBS) and centrifuged again for 10 min at 700 ×*g* at 4°C. The obtained pellets were resuspended in freezing medium [Dulbecco's Modified Eagle Medium (DMEM; Alfagene, Algés, Portugal) supplemented with 10% fetal bovine serum (FBS; Alfagene, Algés, Portugal), 10% dimethyl sulfoxide (DMSO; Sigma-Aldrich, St Louis, MO, USA), 1% of each penicillin, and streptomycin antibiotics (Alfagene, Algés, Portugal)]. The cell suspension was adjusted based on cell counts performed using a Neubauer Chamber under an Olympus BX45 microscope (Olympus Corporation, Tokyo, Japan) ensuring the recommended number of 10^4^ cells per gel for the comet assay. The careful counting and adjustment of the cell suspension ensured that each gel contained the same number of cells, resulting in a consistent number of comets for analysis, with each comet corresponding to the DNA from an individual cell placed in the gel. The samples were then placed in a controlled-rate freezing apparatus (decreasing temperature approximately 1 °C per min) in the −80°C freezer, with enough amount for 2 gels per slide.

### Alkaline comet assay

We used the alkaline comet assay to assess DNA SSB and DSB of healthy individuals and breast cancer patients at two time points of blood collection. The comet assay was essentially conducted as described by Collins et al.^36^ Frozen PBL were thawed, immediately centrifuged for 10 min at 400 ×*g* at 4°C and washed in cold PBS. Each experiment included both a positive control, in which cryopreserved lymphocytes from a healthy individual were exposed to 10 μM hydrogen peroxide (H_2_O_2_) for 5 min on ice (resulting in pronounced DNA damage with 30–40% DNA in tail), and a negative control, using the same unexposed cryopreserved lymphocytes. These controls were processed under identical conditions as the test samples. Cell concentration was adjusted by adding cold PBS to obtain an optimal cell number for an assay of two gels per slide. Prepared cell suspension was mixed with 0.8% low melting point agarose (LMP; Sigma-Aldrich, cat. no. A9414), placed on pre-coated microscope slides, covered with cover slips, and kept for 5 min at 4°C. Then the cover slips were removed, and the slides with cells embedded in agarose were immersed for 1-3 h in lysis buffer solution at 4°C [2.5 M sodium chloride (NaCl), 0.1 M EDTA, 0.01 M Tris-base, pH 10 and 1% Triton X-100], to remove membranes, soluble cytoplasmatic and nuclear components, to obtain nucleoids (supercoiled DNA forming loops). After lysis, the slides were incubated in the electrophoresis solution/alkaline solution [0.3 M sodium hydroxide (NaOH), 1 mM EDTA], for 30 min at 4°C, to denature DNA and then electrophoresis was carried out (at 1 V/cm for 20 min). After electrophoresis, the microscope slides were washed by immersion for 10 min at 4 °C in PBS and then for a further 10 min in deionized water. They were then placed horizontally to dry at room temperature overnight.

### Scoring comets and calculating percentage of DNA in tail

Gels were stained with 25 μL of DAPI (4′,6-diamidine-2-phenylindol dihydrochloride, 1 μg/mL in water) and covered with a cover slip before microscopic analysis. For visualization, a fluorescence microscope (Olympus, Tokyo, Japan) equipped with a filter combination for DAPI and a magnification of 400 × was used. A total of one hundred comets on each gel were visually scored as belonging to one of five classes according to the tail intensity. Comets were selected systematically across the gel in a zigzag pattern, avoiding re-scoring or selecting comets near the edges or areas with imperfections to ensure unbiased and consistent scoring. Each comet class was given a value between 0 and 4: (0) = undamaged and (4) = maximum damage.^37^ The cumulative score for each gel was derived from the sum of the 100 comets assessed, resulting in a scale of 0 to 400 arbitrary units (AU). The levels of DNA damage are expressed as a percentage of DNA in the tail, which corresponds to the portion of fragmented DNA that migrates into the tail during electrophoresis, reflecting the extend of DNA breaks in each cell. This percentage is calculated by dividing the AU value by 4, following the method described by Azqueta, et al.^38^ This parameter is the most widely accepted measure of DNA damage among researchers using this technique. To calculate the variation in DNA damage over time, the percentage of DNA in the tail at t0 (pre-treatment) is subtracted from the percentage at t1 (post-treatment), with the resulting Δt value representing the increase in DNA damage after treatment.

### Statistical analysis

Values are presented as mean ± SD (standard deviation) to provide a clear and detailed view of the distribution of data, facilitating comparison and interpretation.

For comparisons between different groups the Kruskal-Wallis test was applied. A Chi-square test was performed to analyse the association of sex, tobacco, and alcohol consumption between the distinct groups. To determine the correlation between the variables, clinical parameters and DNA damage levels, a Spearman's rank correlation and a Mann-Whitney test was used. To compare the same variable at different time points the paired samples t-test was used. Statistical analyses were performed using software Statistical Program for Social Sciences (SPSS) version 27.0.

## Results

### Characteristics of the study population

Characterization of patients and controls are shown in Table 1. The sample was divided into two groups, the control group (healthy individuals) and breast cancer patients. Information of some DNA damage factors, such as smoking and alcohol habits, body mass index (BMI), age, and sex was obtained by consulting the clinical files of pre-treatment medical sessions. Regarding BMI, smoking habits, and alcohol consumption, a statistically significant difference was discovered between breast cancer and the control groups, with *p* values of 0.035 for BMI, 0.034 for smoking habits, and 0.000 for alcohol consumption (see Table 1 for details). Five breast cancer patients were found to be associated with hereditary cancers markers with pathogenic variants in genes: the Ataxia-telangiectasia mutated gene (*ATM*) - two patients, RAD 51 paralog C gene (*RAD51C)*, encodes Rad1 protein that acts in DNA repair and homologous recombination, Checkpoint kinase 2 gene (*CHEK2),* a tumor suppressor gene that encodes a serine/threonine kinase, and *BRCA1*. It is known that alterations in these genes play a crucial role in DNA repair and increase the risk of developing breast cancer.^39^ Table 1 also shows the number of patients in each clinical stage at diagnosis, as well as the type of treatment they received: CT, C-RT, and RT.

**Table 1.** Characterization of breast cancer patients and control groups.

			Total	Groups		*p*
		*n*	Mean ± SD	Control	Breast cancer	
	Sporadic			31	18	
	Hereditary				5	
	Not studied				35	
Age		89	57.55 ± 12.53	58.29 ± 13.82	57.16 ± 11.89	*0*.*906*
BMI		89	27.46 ± 4.05	25.83 ± 3.07	28.32 ± 4.25	*0*.035*
		*n (%)*	*n (%)*			
Sex	Female	78 (*87.6)*	20 (*64.5)*		58 (*100)*	*0*.000**
	Male	11 (*12.4)*	11 (*35.5)*		0 (*0.0)*	
Smoking	Never	57 (*66.3)*	15 (*48.4)*		42 (*77.8)*	*0*.034**
	Former	18 (*20.9)*	11 (*35.5)*		7 (*13.0)*	
	Smoker	11 (*12.8)*	5 (*16.1)*		5 (*9.3)*	
Alcohol Consumption (g/day)	No	31 (*36.1)*	10 (*54.5)*		21 (*38.2)*	*0*.000**
	≤ 20 g/day	48 (*55.8)*	15 (*36.4)*		33 (*60.0)*	
	> 20 g/day	7 (*8.1)*	6 (*9.1)*		1 (*1.8)*	
Clinical Stage	I				7 (*12.1)*	
	II				35 (*60.3)*	
	III				16 (*27.6)*	
Treatment	CT				45 (*78.9)*	
	C-RT				12 (*21.1)*	
	RT				0 (*0.0)*	

Statistical meaning: *p values were analysed by Kruskal-Wallis test. **p values were analysed by Chi-square test, data are shown as mean ± S.D.

### DNA damage levels at t0

**Figure 1.**
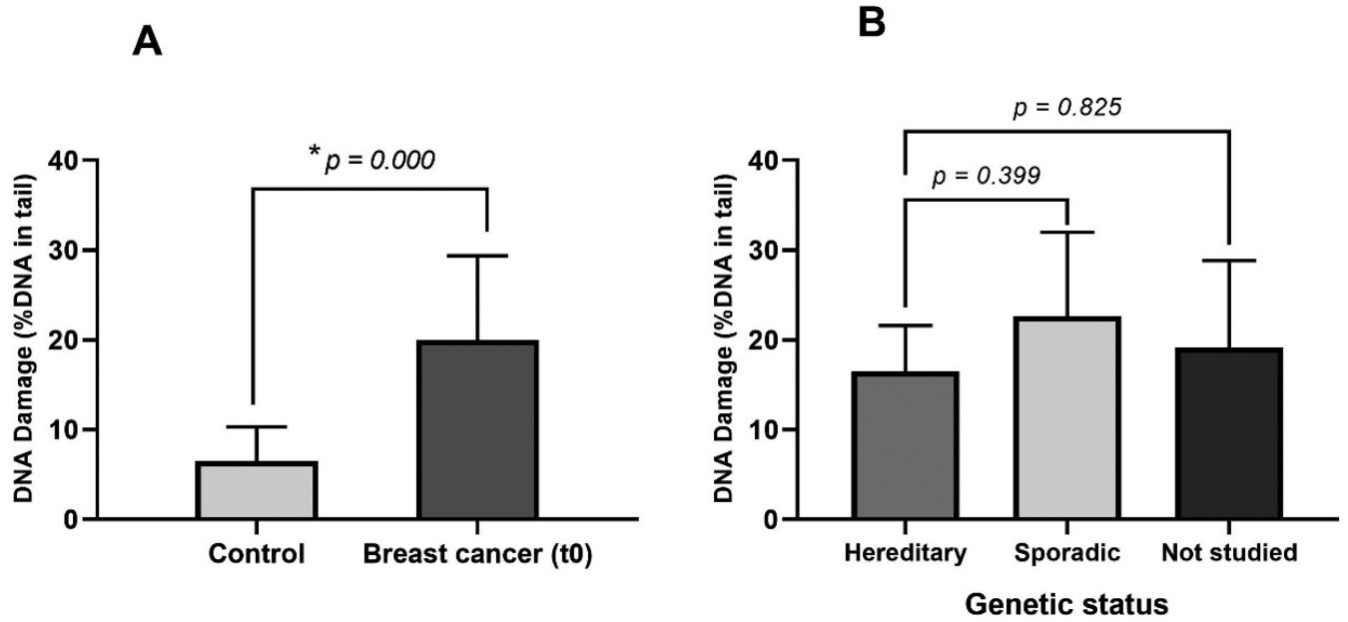
DNA damage levels of A) breast cancer patients and healthy controls, and B) breast cancer patients divided into sporadic, hereditary, and not studied cases. Mean values are shown with SD. Statistical significance was determined using the Kruskal-Wallis test, with *p-values *≤ 0.05 considered significant (*).

Figure 1 shows the differences between control group and breast cancer patients regarding DNA damage levels (DNA SSB and DSB). We observed that mean values of DNA in tail percentage of breast cancer's PBL at t0 (20.23 ± 9.40) were significantly higher in comparison to control group (9.09 ± 5.41), with *p *= 0.003. In addition, we found that the confirmed hereditary cases of breast cancer did not show significantly different DNA damage levels (16.5 ± 5.1) compared to the sporadic cases (22.6 ± 9.4, *p *= 0.399) and the cases that were not studied (19.1 ± 9.7, *p *= 0.825).

To better understand whether the high levels of DNA damage in cancer patients were related to factors that could affect DNA integrity, we applied Spearman and Mann-Whitney statistical correlation tests between these variables and the DNA damage results obtained by the comet assay. As shown in Table 2, we observed that there was no statistical correlation between DNA damage and any of the studied variables. Specifically, no significant correlation was found between DNA damage and age (*p *= 0.484), or BMI (*p *= 0.681). Additionally, smoking status (*p *= 0.574) and alcohol consumption (*p *= 0.954) also showed no significant association with DNA damage levels.

**Table 2.** Correlations between DNA damage levels and biomarkers of damage.

Variables		Control		Breast cancer		
		rho	*p*	*rho*	*p*	
Age		*−0.030*	0.871	*−0*.*094*	*0*.*484*	Spearman's rank correlation
BMI		*−0.306*	0.100	*0*.*056*	*0*.*681*	
Sex	Female		0.836			Mann-Whitney test
	Male					
Smoking	Never		0.471		*0*.*574*	
	Former					
	Smoker					
Alcohol Consumption (g/day)	No		0.290		*0*.*954*	
	≤ 20 g/day					
	> 20 g/day					

The correlation is significant if *p *≤ 0.05.

**Figure 2.**
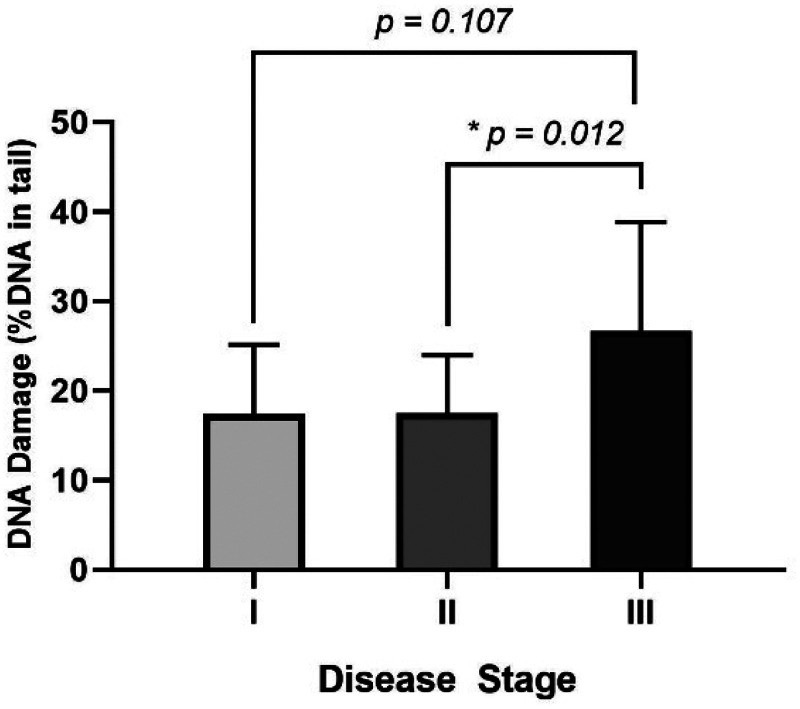
Levels of DNA damage of breast cancer patients at disease stages I, II and III. Statistical significance was determined using the Kruskal-Wallis test, with *p-values *≤ 0.05 considered significant (*).

To evaluate the predictive potential of DNA breaks for disease staging, we categorized the patients of this study based on their stage at diagnosis. Figure 2 shows DNA damage levels of breast cancer patients at each disease stage. We observed an increase in DNA damage levels among breast cancer patients in stage III (26.70 ± 12.15) compared to those at stage I (17.39 ± 7.70) and II (17.46 ± 6.51), with a statistically significant difference particularly between stages II and III (*p *= 0.012).

### DNA damage levels at t1

**Figure 3.**
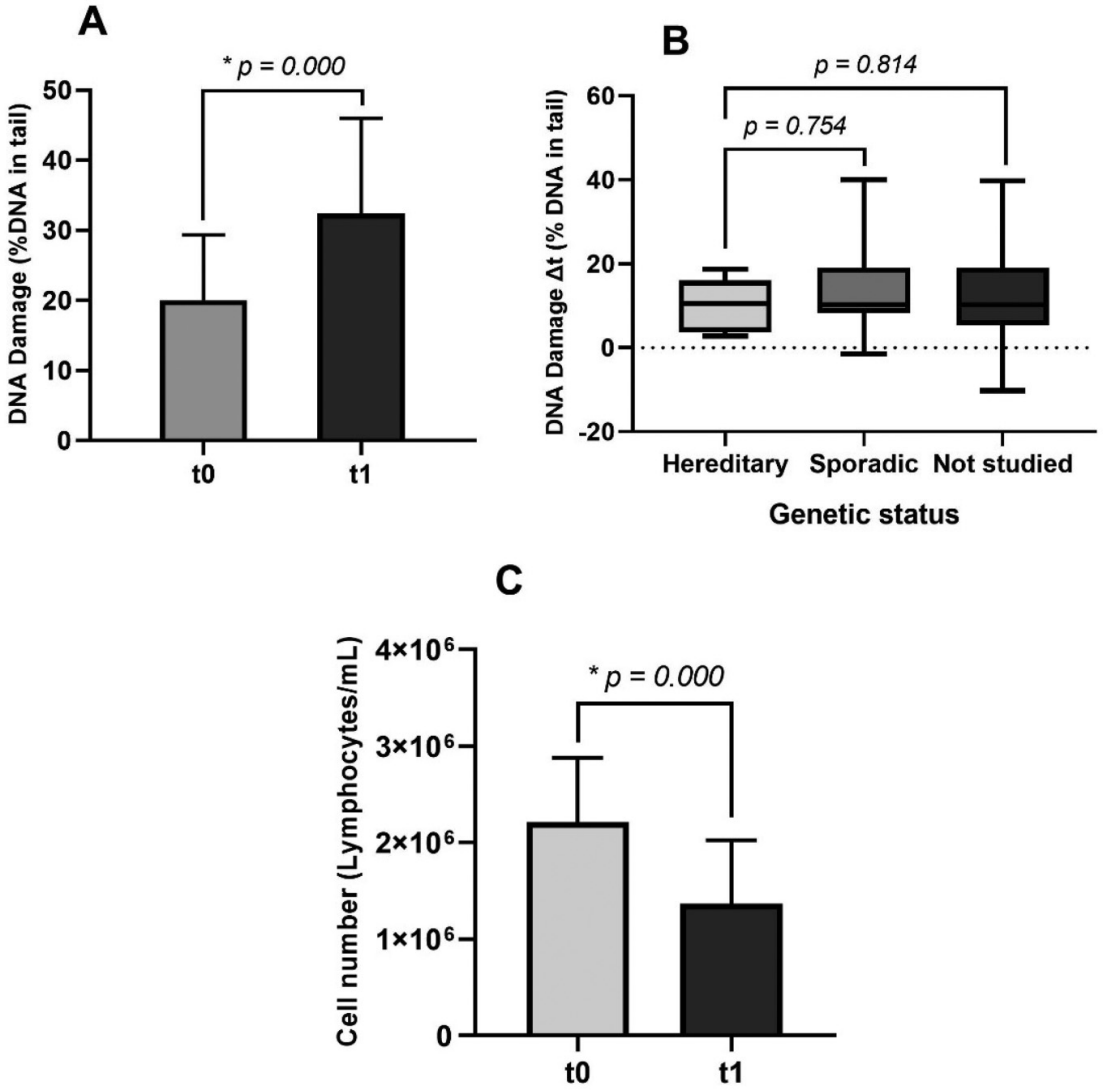
DNA damage levels at t0 and t1 of breast cancer patients. (B) Variations in DNA damage levels between t0 and t1 divided into sporadic, hereditary, and not studied cancer cases. (C) Number of PBLs at t0 and t1 of breast cancer patients. Significance of the data in graphs (A) and (C) were analyzed by paired samples t-test, and data in graph (B) was analyzed by the Kruskal-Wallis test, with *p-values *≤ 0.05 considered significant (*).

After treatment administration (t1), there was a significant increase in DNA damage levels (*p *= 0.000), by a rise to 32.41 ± 13.59% DNA in tail (Figure 3A). Furthermore, we found that the variation in the percentage of DNA in the tail from t0 to t1 (DNA damage Δt) was not significantly (*p *= 0.754) difference between sporadic and hereditary cancer cases (Figure 3B). Simultaneously, a statistically significant decrease (*p *= 0.000) in the number of PBL in breast cancer patients was observed, from 2.21 × 10^6^ ± 6.68 × 10^5^ to 1.37 × 10^6^ ± 6.57 × 10^5^ /mL (Figure 3C). Despite this, there was no statistically significant correlation between the degree of lymphocyte depletion and the increase in DNA damage levels (*p *= 0.572). However, our results consistently demonstrate both a reduction in circulating lymphocytes after treatment and an increase in DNA damage in nearly all patients.

### CA 15-3 and correlation with DNA damage levels

**Figure 4.**
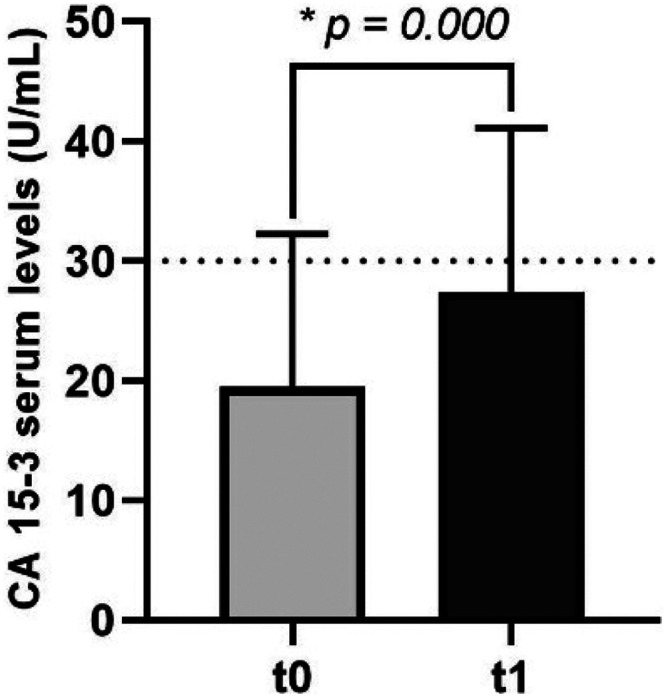
Ca 15-3 serum levels at t0 and t1 treatment administration to breast cancer patients. The reference values of CA 15-3 values in serum are lower than 30 U/mL.^40^ Significance of the data was analysed by paired samples t-test, with *p-values *≤ 0.05 considered significant (*).

Moreover, to provide a comparison with a clinically established non-invasive marker, we analyzed the serum levels of the tumor marker CA 15-3 at both t0 and t1. We also correlated the values of CA 15-3 with those of DNA damage to evaluate whether the assessment of DNA damage levels could offer enhanced diagnostic and monitoring capabilities. Firstly, it was found that most breast cancer patients (88%) had serum levels of CA 15-3 below the established cut-off value of 30 U/mL,^40^ as defined in early studies on this biomarker and consistent with its reference values.^41–43^ The levels assessed at t0 were 19.79 ± 13.28 U/mL. After treatment administration, there was a statistically significant increase (*p *= 0.000) in levels of CA 15-3 (28.24 ± 15.74 U/mL) in breast cancer patients (Figure 4).

Furthermore, it was observed that the variations in the levels of CA 15-3 biomarker between t0 and t1 (CA 15-3 Δt) did not show a statistical correlation (*p *= 0.992) with DNA damage Δt, as indicated in Table 3. Notably, these variations did not demonstrate uniform responsiveness to treatment.

**Table 3.** Correlations of the difference between t0 and t1 DNA damage levels (DNA damage Δt) and difference between t0 and t1 of CA 15-3 biomarker serum levels (CA 15-3 Δt).

	DNA damage Δt		
	*rho*	*p*	
CA 15-3 Δt	0.001	0.992	Spearman's rank correlation

The correlation is significant if *p *≤ 0.05.

### DNA damage levels across treatment groups

**Figure 5.**
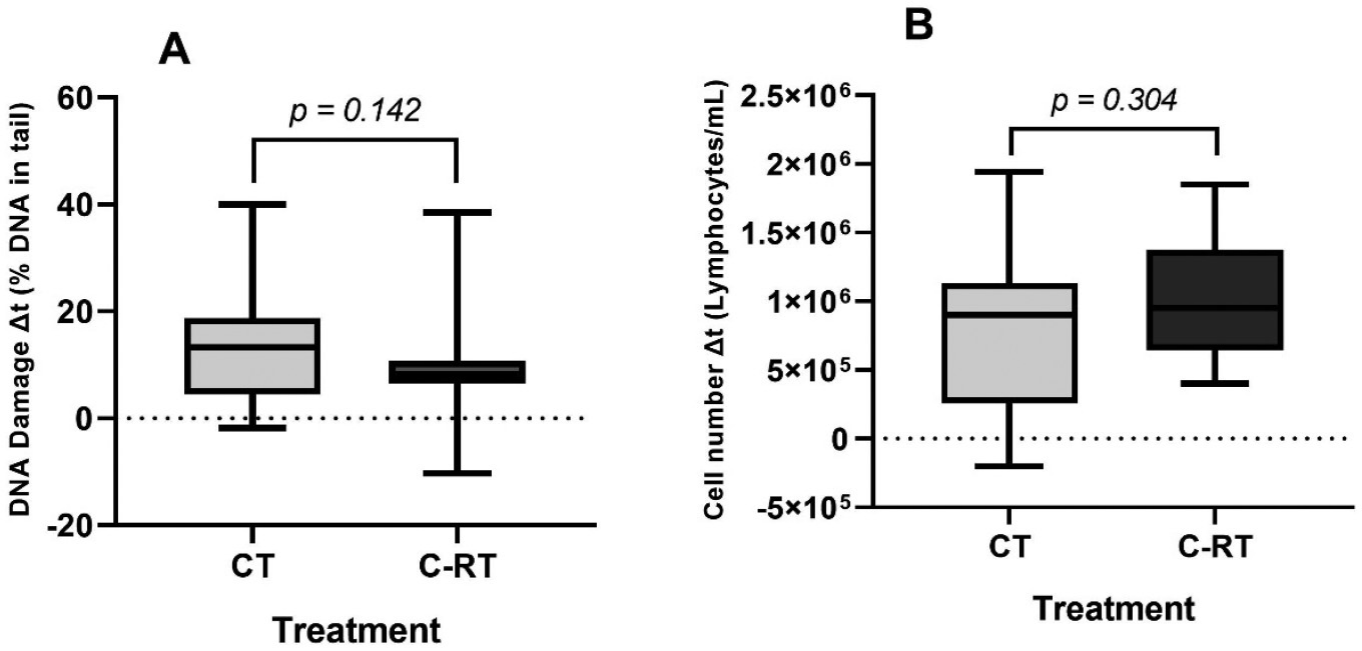
Differences between t0 and t1 (Δt) of A) DNA damage levels and B) number of PBL of breast cancer patients undergoing CT or C-RT. Significance of the data was analysed by Mann-Whitney test, with *p-values *≤ 0.05 considered significant (*).

Patients were divided into groups: CT and C-RT, to assess the individual effects on PBL of each type of treatment applied. Figure 5 illustrates that although CT patients exhibited a higher variation in DNA damage levels and a lower variation in PBL count compared to C-RT patients, no statistically significant difference was observed between the two types of treatment *(p *= 0.142 for DNA damage variation, *p *= 0.304 for PBL count variation).

**Figure 6.**
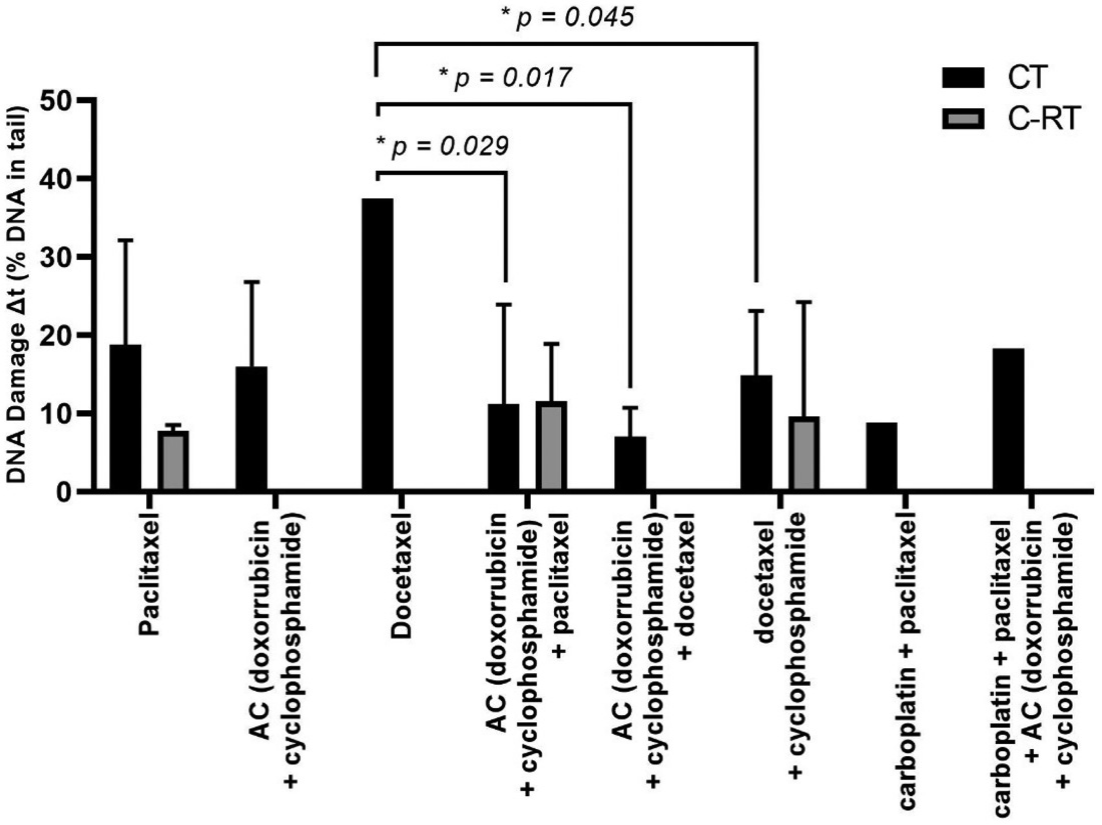
Differences between t0 and t1 (Δt) in DNA damage levels of breast cancer patients undergoing combined drugs. Significance of the data was analysed by Pairwise comparisons, with *p-values *≤ 0.05 considered significant (*).

To better understand the impact of chemotherapy drugs, administered individually or in combination, on DNA damage levels of PBL, we categorized patients receiving CT into groups based on the administered drugs. Additionally, we evaluated the statistical differences of DNA damage levels between these distinct groups. Figure 6 illustrates the variations in levels of DNA damage induced by each drug combination, both with and without radiotherapy. It was observed that the application of the drug docetaxel alone induced a significantly higher variation in DNA damage levels compared to drug combinations: AC (doxorubicin and cyclophosphamide) combined with paclitaxel (*p *= 0.029); AC combined with docetaxel (*p *= 0.017); and docetaxel combined with cyclophosphamide (*p *= 0.045). However, no statistical association was found between the other different drug combinations.

## Discussion

The present study aimed to assess the potential of DNA damage of PBL as a biomarker for genome instability in breast cancer, examining its relevance in diagnostic staging and predicting treatment outcomes, in both sporadic and hereditary cases. For this purpose, the levels of DNA strand breaks were evaluated by simple comet assay, before and after treatment exposure. PBL were used as the surrogate tissue due to their capacity to circulate in the body for extended periods, during which they accumulate DNA alterations resulting from various exposures experienced throughout their lifespan, and reflect a similar damage of cells of target tissue.^44^ To explore the potential of DNA damage assessment as a marker of breast tumor severity, we also investigated the correlation between basal levels of DNA strand breaks and the stage of the disease at diagnosis. In addition, we also studied its correlation with serum CA 15-3 levels and the viable PBL count, some clinical parameters that may be associated with treatment response. In the comprehensive analysis of our study cohort significantly divergent trends were observed between the breast cancer cohort and the control group in relation to the variables of BMI, smoking habits, and alcohol consumption. These findings align with the well-established understanding that lifestyle habits can contribute to the development of breast cancer.^45–47^ Therefore, it is reasonable to expect that these variables are linked to the occurrence of this disease.

Concerning genomic instability in cancer, numerous studies have explored the implications of DNA integrity failure in breast cancer development and its predictive capacity in post-treatment monitoring.^34,48–51^ Our findings consistently revealed a significant increase in DNA damage of breast cancer patients’ PBL prior to treatment compared to the control group. The DNA damage basal levels observed in healthy participants are attributed to natural cellular processes and exposure to environmental factors, which cause DNA damage and repair activities, as has been studied by other authors.^52^ Additionally, we found no statistically significant differences in the SSB and DSB levels between breast cancer cases with mutated and non-mutated repair genes, with DNA damage levels in hereditary cases being comparable to, and in some instances even lower than those observed in sporadic cases. However, since the mutated patients carried mutations in key DNA repair genes, it would expect them to exhibit higher levels of DNA damage compared to the non-mutated group, even with only five hereditary cases in cohort. The lack of significant differences suggests that there may be external regulation of these repair mechanisms^53^ that could disrupt cell's repair activity,^54^ even in patients without mutations in these critical genes, which may contribute to breast cancer development. These findings suggest that phenotypic assessments, such as DNA damage measurement, may offer a more functional insight into cellular responses to damage than solely genotypic information. Some authors have sought to determine whether the increase in DNA damage is influenced by some factors and associated with cancer itself. Some have noted that lifestyle habits, like smoking, and age showed no correlation with the observed DNA damage in untreated breast cancer patients.^48^ Our study suggests that the increased DNA damage observed does not appear to be related to alcohol consumption, age, BMI, or sex. These findings may support the insight that DNA damage is independent of these factors and may be a fundamental driver in the initiation and development of breast cancer. After treatment, a significant increase in DNA strand breaks levels was observed, coinciding with a reduction in PBL number among these patients, which may suggest treatment-induced extensive DNA fragmentation potentially triggering cell death. It is important to note that the second sample collection occurred a few days after the end of the last treatment session, coinciding with the day of their post-treatment follow-up appointment. This timing allowed for the repair system to potentially respond, ensuring that the data measured at t1 reflected the DNA damage levels persisting after the treatment. While these results were unsurprising, given that the DNA molecule serves as the primary target for the treatment employed,^55^ they highlight a critical aspect: DNA damage serves not only as an indicator of cell death due to excessive treatment-related DNA damage but also provides functional insights into the cellular response to treatment, since most cancer therapies inherently affect DNA. Surviving cells that were unable to repair the damage exhibit measurable residual damage at t1, which can become especially relevant in cases where imaging may not capture early changes in treatment response, allowing for a more timely evaluation of treatment efficacy. Moreover, our findings indicate that patients with mutated repair genes responded similarly to treatment compared to those without mutations. This highlights the possibility of compensatory mechanisms in mutated cases or ineffective external regulation of DNA repair processes in non-mutated cases, suggesting that environmental factors may significantly influence DNA repair activity among the subjects in this study. Consequently, this reaffirms its potential as a valuable diagnostic biomarker, as well as its promising predictive capacity for post-treatment monitoring.

To further explore the relevance of DNA damage assessment in the clinical setting of breast cancer, we examined the correlation between DNA damage levels, disease stage, and CA 15-3 serum levels. This cancer protein is one of the few non-invasive biomarkers commonly used in clinical practice, and it is also the primary non-invasive method for monitoring the patients at the hospital where our research was conducted. This allowed us to compare its diagnostic and monitoring capabilities with those of the central method under study, the assessment of DNA damage, which is also a non-invasive approach. Our findings suggested a direct and positive connection between disease stage and the DNA damage levels detected at diagnosis, as already reported.^56^ This observation implies that evaluating DNA damage might be useful as an adjunct criterion for diagnosing and assessing the aggressiveness of breast cancer. In contrast, our observations revealed no statistical correlation between the variability of DNA damage levels and the variation of CA 15-3 serum levels, suggesting that there may be a distinct response to treatment between these variables. Interestingly, prior research has delved into the evaluation of DNA integrity assessed through circulating DNA fragments as a diagnostic and monitoring tool for breast cancer in comparison with conventional cancer proteins, such as CA 15-3.^57^ Nevertheless, exploring the relationship between the DNA damage levels inside the circulating cell and this cancer antigen represents a new approach not previously addressed. Simultaneously, we also observed that serum CA 15-3 levels at t0 were below the threshold point in majority of breast cancer patients. Following treatment administration, these values increased significantly, suggesting tumor cells degradation and the release of antigen into the bloodstream. After this, the values exhibited a gradual decline due to the elimination of the antigen from the bloodstream. We also found that this protein exhibited heterogeneous fluctuations across all patients, whereas the DNA damage levels consistently varied in a uniform manner in response to treatment among the entire cohort under investigation. Consistent with earlier investigations, our findings substantiate the lack of significance of CA 15-3 in primary breast cancer screening or diagnosis.^58^ Thus, our cumulative evidence indicates that the evaluation of DNA strand breaks plays a more consistent and objective role in both the diagnosis and prompt monitoring of breast cancer treatment.

It was also our goal to assess how treatment choices could differentially impact DNA damage levels and PBL count. The selection of treatment - whether CT or C-RT - in breast cancer is contingent upon various factors, such as cancer stage, biological attributes like hormone receptors and HER2 status, the patient's overall health, and existing medical conditions.^59^ It is known that RT treatment is commonly employed in clinic practice post-surgical excision to eradicate any remaining subclinical disease.^20^ Specifically, the patients included in this study who underwent adjuvant RT received this treatment subsequent to adjuvant CT. Our results indicated that the concurrent use of RT alongside adjuvant CT did not lead to significant variations in the DNA strand breaks basal levels in PBL, suggesting that this genotoxicity marker remains unaffected by the treatment choice. On the other hand, we evaluated the impact of each chemotherapy drug or drug combination on DNA damage levels. Indeed, previous studies have suggested that DNA damage in circulating cells may be used as a valuable biomarker for assessing responses to antineoplastic drugs.^60–62^ However, definitive conclusions remain elusive. Patients’ responses to chemotherapy are known to vary based on several factors, including DNA repair capacity, which can differ due to genetic polymorphisms, pre-existing oxidative stress, and immune function.^63,64^ Although anthracyclines are generally expected to cause more DNA damage due to their potent genotoxic effects, our findings revealed a significantly higher variation in the DNA strand breaks levels in response to the application of docetaxel compared to various drug combinations. This discrepancy could be attributed to the direct impact of docetaxel on cell division, resulting in more significant DNA damage to lymphocytes when administered alone. Moreover, it is important to consider that this particular patient receiving docetaxel was both obese and an active smoker, factors known to increase oxidative stress and inflammation, affecting drug pharmacokinetics and response, which could have contributed to the heightened DNA damage observed.^65,66^ In addition, it is recognized that drug combinations are sometimes employed to achieve synergistic effects, enabling the use of lower doses for each drug, and consequently reducing damage to healthy cells, including lymphocytes.^67^ Despite the limited number of patients and the frequent use of combination therapies, our results suggest that measuring DNA damage is a valuable tool for assessing treatment efficacy, as all patients in this study showed an increase in DNA strand breaks after treatment. This reinforces the notion that this biomarker complements existing imaging guidelines. While imaging remains essential for tracking tumor response, DNA damage assessment can provide critical insights into the underlying cellular response to treatment, helping clinicians evaluate the effectiveness of the therapy more holistically.

Even though this study achieved significant results, some suggestions for improving our conclusions should be considered. The number of subjects does not allow us to make clear comparisons between homogeneous groups of different stages of the disease, type of treatment they underwent and nature of the tumor. It would be necessary to extend the study to a larger number of patients and assess DNA damage at more time points to better understand the role of DNA damage as a biomarker throughout treatment. In addition, given that genome stability depends on DNA repair mechanisms, the data from this study clearly highlights the need to analyze the role of DNA repair mechanisms in the process of tumorigenesis and in the response to treatment.

## Conclusion

The results from the present study significantly underline the potential of DNA damage levels assessment as a biomarker for both sporadic and hereditary breast cancer and for monitoring its treatment, far surpassing the conventional CA 15-3 for this indication. This reinforces the urgency of further exploration and clinical application. Nevertheless, for the comet assay to be employed as a tool for oncologists, this relationship should be validated in a larger patient cohort, and the methods for normalizing and interpreting the data need clarification.
